# Intrastriatal Memantine Infusion Dampens Levodopa-Induced Dyskinesia and Motor Deficits in a Mouse Model of Hemiparkinsonism

**DOI:** 10.3389/fneur.2019.01258

**Published:** 2019-12-05

**Authors:** Masatoshi Ogawa, Yu Zhou, Ryosuke Tsuji, Jiro Kasahara, Satoshi Goto

**Affiliations:** ^1^Department of Neurodegenerative Disorders Research, Institute of Biomedical Sciences, Graduate School of Medical Sciences, Tokushima University, Tokushima, Japan; ^2^Department of Neurobiology and Therapeutics, Institute of Biomedical Sciences, Graduate School of Pharmaceutical Sciences, Tokushima University, Tokushima, Japan; ^3^Parkinson's Disease and Dystonia Research Center, Tokushima University Hospital, Tokushima, Japan

**Keywords:** intracerebral brain infusion, levodopa-induced dyskinesia, memantine, *N*-methyl-D-aspartate receptor, Parkinson's disease

## Abstract

Although the administration of dopamine precursor levodopa remains as the mainstay for the treatment of Parkinson's disease, long-term exposure to levodopa often causes a disabling complication, referred to as levodopa-induced dyskinesias. Therefore, the development of new therapeutic interventions to dampen levodopa-induced dyskinesias and parkinsonian motor deficits is needed in the treatment of Parkinson's disease. Intracerebral brain infusion has the merit of being able to specifically deliver any drug into any brain part. By using an intracerebral infusion system equipped with implantable, programmable, and refillable pumps, we show herein that continuous intrastriatal administration of memantine (MMT), which is a non-competitive *N*-methyl-D-aspartate receptor antagonist, attenuates levodopa-induced dyskinesias and parkinsonian signs in 6-hydroxydopamine-lesioned hemiparkinsonian mice that received daily levodopa treatment. Corroborating the general thought that overactivation of the striatal *N*-methyl-D-aspartate receptor function might generate levodopa-induced dyskinesias and parkinsonism, our results suggest that a continuous intrastriatal MMT infusion can be beneficial for the management of Parkinson's disease with levodopa-induced dyskinesias. Our study also provides indications for the prototypic use of pharmacological deep-brain modulation through intracerebral infusion systems for treating medically intractable movement disorders.

## Introduction

The administration of the dopamine precursor levodopa is a powerful tool for treating Parkinson's disease; however long-term exposure to levodopa often provokes abnormal involuntary movements (AIMs), referred to as levodopa-induced dyskinesias (LIDs) (1). LID is a major cause of disability that occurs in >80% of Parkinson's disease patients after 5 years of daily levodopa treatment (2). Although electrical deep brain stimulation can produce a striking impact on Parkinson's disease with LIDs, its therapeutic efficacy for the quality of life has usually fallen below preoperative levels during long-term follow-up (3). Therefore, the development of alternative or adjunct therapeutic interventions for overcoming both LIDs and “OFF”-period symptoms is a pressing challenge in the treatment of Parkinson's disease.

Parkinsonian symptoms result from striatal dysfunction caused by an imbalance between dopamine and glutamate transmissions (4, 5). The dysregulation of corticostriatal glutamatergic inputs leading to maladaptive synaptic plasticity in the striatum is also a well-documented process underlying LID genesis (6). At the striatal postsynaptic level, the interactions of D_1_-type dopamine receptors with glutamate receptors, particularly *N*-methyl-D-aspartate (NMDA) receptors and metabotropic glutamate receptors, appear to be especially relevant (6–9). Accordingly, oral administration of compounds targeting NMDA receptors or specific subtypes of metabotropic glutamate receptors (mGluR4 and mGluR5) has been challenged in clinical practice (8). Currently, the oral administration of amantadine, which is a non-competitive NMDA receptor antagonist, represents the only recommended adjunct therapy for reducing LIDs in patients with Parkinson's disease (9). However, its low therapeutic efficacy and various adverse effects have limited its clinical use.

Applying the intracerebral brain infusion (iCBI) technique for brain disorder treatment has a long history in experimental and clinical studies on Parkinson's disease [for examples, see (10–12)]. Interestingly, Whone et al. (13) recently reported the clinical use of a new and sophisticated drug delivery system for intermittent intraputamenal infusion of the glial cell line-derived neurotrophic factor in patients with Parkinson's disease. The present study was undertaken to examine if the continuous intrastriatal infusion of memantine (MMT), which is a non-competitive NMDA receptor antagonist and clinically used for the treatment of dementia (14), through an iCBI system could attenuate levodopa-induced AIMs and parkinsonian signs in mice with hemiparkinsonism caused by 6-hydroxydopamine (6-OHDA) lesions. Our results suggest that continuous intrastriatal MMT infusion may represent a new therapeutic tool in treating intractable LIDs associated with Parkinson's disease.

## Materials and Methods

### Animals

Adult male C57BL/6 mice aged 8–9 weeks (Nihon SLC, Hamamatsu, Japan) were used. The mice were housed in a controlled environment (25 ± 1°C, 50 ± 10% humidity, and 12-h light/dark cycle) with access to food and tap water *ad libitum*. All experimental procedures were approved by the Institutional Animal Care and Use Committees of Tokushima University, Japan.

### Nigrostriatal 6-OHDA Lesion

**Figure 1:**
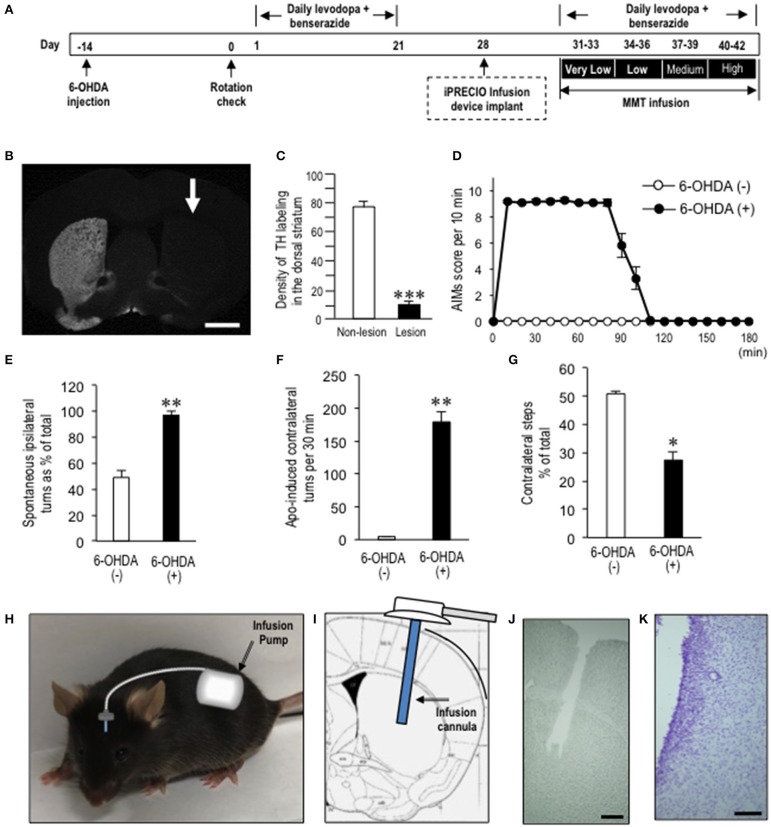
Generation of dyskinetic mice for the intracerebral brain infusion (iCBI) experiments. **(A)** Timeline of experimentations (see “Materials and Methods” section). Dyskinetic mice were implanted with programmable intracerebral brain infusion (iCBI) systems equipped with iPRECIO^TM^ micro infusion pumps at day 28. After a recovery of 3 days, they restarted receiving daily levodopa/benserazide treatments for the next 12 days. Simultaneously, MMT-treated dyskinetic mice received continuous intrastriatal infusion of MMT 0.28 ng/μl at a flow rate of 1.0 μl/h for the first 3 days (days 31–33), MMT 1.12 ng/μl at a flow rate of 1.0 μl/h for the next 3 days (days 34–36), MMT 4.48 ng/μl at a flow rate of 1.0 μl/h for the next 3 days (days 37–39), and MMT 17.93 ng/μl a flow rate of 1.0 μl/h for the last 3 days (days 40–42). In parallel, PBS-treated dyskinetic mice received PBS under the same protocol. AIM scoring tests for MMT- or PBS-treated mice were done at days 33, 36, 39, and 42. **(B)** A representative photomicrograph of the striatum stained for tyrosine hydroxylase on the non-lesioned and lesioned (arrow) sides in mice with 6-OHDA-induced lesions. Scale bar = 2 mm. **(C)** Quantification of the mean staining intensity for tyrosine hydroxylase (TH) in the dorsolateral striatum on the non-lesioned (non-lesion) and lesioned (lesion) sides. In the Y-axis, the averaged pixel intensities in the measured area were expressed with arbitrary units. Data are means ± SEM (*n* = 7). Paired two-tailed *t-*test: ***P < 0.001 vs. non-lesion. **(D)** Time course of the abnormal involuntary movements (AIMs) scored every 10 min over a period of 180 min after the last levodopa administration in naïve mice and 6-hydroxydopamine (6-OHDA)-lesioned mice. Data are means ± SEM at each time point (naïve mice, *n* = 10; 6-OHDA-lesioned mice, *n* = 14). **(E)** Spontaneous rotations ipsilateral to 6-OHDA-lesioned side percent of total in mice with or without 6-OHDA lesions. 6-OHDA (–) (*n* = 6); 6-OHDA (+) (*n* = 6). Data are means ± SEM. **P < 0.01 vs. 6-OHDA (–); Mann-Whitney U-test. **(F)** Counts of apomorphine-induced contralateral turns per 30 min in mice with or without 6-OHDA lesions. 6-OHDA (–) (*n* = 6); 6-OHDA (+) (*n* = 6). Data are means ± SEM. **P < 0.01 vs. naïve controls; Mann-Whitney U-test. **(G)** Counts of hind limb steps. Steps of the contralateral hind limb as % of the total count in the naïve controls (*n* = 6) and Hemi-PD mice (*n* = 6). Data are means ± SEM. *P < 0.05 vs. naïve; Mann-Whitney U-test. **(H–K)** Schematic representation of the iCBI system, in which the pump and tubing were implanted completely under the skin **(H)**. The infusion cannula implanted into the dopamine-depleted striatum **(I)**, together with the image of striatum after fixation and removal of the cannula **(J)**, and with a Nissl-stained image **(K)**. Scale bars show 0.5 mm in **(J)** and 100 μm in **(K)**.

Under anesthesia with N_2_O and isoflurane (Sigma-Aldrich, St. Louis, MO, USA), the mice received an intraperitoneal (i.p.) injection of desipramine hydrochloride (an inhibitor of noradrenaline transporter, 1 mg/kg dissolved in 0.9 % saline, Wako, Osaka, Japan) and pargyline hydrochloride (an inhibitor of monoamine oxidase, 0.2 mg/kg dissolved in 0.9% saline, Sigma-Aldrich, St. Louis, MO, USA). We used these inhibitors for uptaking 6-OHDA selectively in brain dopaminergic neurons. After 15 min, they received stereotaxic injections of 6-OHDA HCl (Sigma-Aldrich; 3 μg dissolved in 0.2 μl of 0.9% saline containing 0.2% ascorbic acid) targeted into the medial forebrain bundle (MFB) on the right side. The target coordinates (AP = −1.2, L = +1.1, DV = +5.0) were according to the mouse brain atlas (15). After a recovery of 2 weeks, the 6-OHDA-lesioned mice, which showed over 80% of the spontaneous ipsilateral rotation and apomorphine (0.5 mg/kg; Sigma-Aldrich) injection-induced contralateral rotation (rotation check) at day 0 (Figure 1A), were processed for further studies.

### Levodopa/Benserazide Treatments

Mice received intraperitoneal injections of levodopa (Sigma-Aldrich; 15 mg/kg) dissolved in 0.9% saline containing 0.5% carboxymethyl cellulose at 20 min after i.p. injections of benserazide HCl (Sigma-Aldrich; 12.5 mg/kg) dissolved in 0.9% saline.

### Assessment of AIMs

AIM scoring was performed after the last levodopa injection for 1 min every 10 min over a period of 150–180 min, as per our previous report (16). For the evaluation, each mouse was placed in a 12-cm-diameter glass cylinder. The following subtypes of AIMs were examined: axial (twisted posturing of the neck and the upper body toward the contralateral side), forelimb (jerky movements of the contralateral forelimb, and/or grabbing movement of the contralateral paw), and orolingual (jaw movements and tongue protrusion toward the contralateral side). Each subtype was scored as follows: 0: absent; 1: occasional; 2: frequent; 3: continuous; and 4: continuous, not interrupted by sensory stimuli.

### Assessment of the Rotational Behaviors

Spontaneous rotational behaviors were assessed by measuring systemic turns ipsilateral to the 6-OHDA lesion (17) 6 h after the last levodopa administration. Apomorphine-induced rotations contralateral to the 6-OHDA lesion were also measured 30 min after the apomorphine (0.5 mg/kg) administration.

### Assessment of Hind Limb Stepping

Video-based analysis of the hind limb stepping in mice was performed as reported previously (18). Mice were placed in 600-ml beaker, and the spontaneous steps of their hind limbs were video-recorded from the bottom for 5 min. Each hind limb was counted with a playback speed slowed to 0.3–0.5× using VLC media player. Total hind limb steps were summed for both the ipsilateral (intact) and contralateral (impaired) to the lesion side, and the number of contralateral steps was calculated as % of the total.

### Placement of iCBI Devices and MMT Administration

The iCBI devices equipped with the iPRECIO^TM^ programmable micro infusion pumps (Model SMP-300, Primetech Co., Tokyo, Japan) were implanted in the mice under anesthesia with isoflurane (Sigma-Aldrich). The infusion cannulas were implanted into the dopamine-depleted striatum at the stereotaxic coordinates: AP = +0.3; L = +2.3; and DV = +3.0. MMT HCl (Wako) was dissolved in 0.01 M phosphate-buffered saline at pH 7.2 (PBS), then applied for the infusion pumps. The used concentrations of MMT were determined by the solubility to PBS of MMT.

### Immunohistochemistry and Digital Imaging

Immunostaining procedures and microscopic imaging were previously described (19). The mice (*n* = 7) were intraperitoneally injected with a lethal dose of pentobarbital (Sigma-Aldrich), and transcardially perfused with cold saline, followed by cold 4% paraformaldehyde in 0.1 M phosphate buffer (pH 7.2). The brains were removed, postfixed overnight in the same fixative at 4°C, and stored in a 10–30% sucrose gradient in 0.01 M phosphate buffered saline (pH 7.2) at 4°C for cryoprotection. Sections were cut on a cryostat at 16-μm thickness and stored in PBS containing 0.05% NaN_3_ until use. After blocking endogenous peroxidase activity, the free-floating sections were incubated in PBS containing 3% bovine serum albumin (PBS-BSA) for 60 min. They were then incubated in PBS-BSA with a rabbit polyclonal antibody tyrosine hydroxylase (TH) (1:200,000) (19) for 18 h. The bound antibody was detected using the Histofine Simple Stain Kit (Nichirei, Tokyo, Japan) and the tyramide signal amplification system with Cyanine3 (Perkin Elmer, Shelton, CT, USA). For Nissl staining, sections were stained with 0.1% cresyl violet acetate (Wako) dissolved in distilled water. Digital microscopy images were captured using an Olympus BX51 microscope (Olympus, Tokyo, Japan), imported into Adobe Photoshop CS4, and digitally processed for adjustments of contrast, brightness, and color balance.

### Statistical Analysis

All analyses were expressed as group mean ± SEM. We used paired two-tailed *t-*test or Mann-Whitney U-test for two-group comparisons. Multiple comparisons were analyzed using analyses of variance (ANOVA), followed by Bonferroni's *post-hoc* tests for pairwise comparisons. Statistical analyses were performed using GraphPad Prism 7 Software (GraphPad Software Inc., San Diego, CA, USA). *P* < 0.05 were considered statistically significant.

## Results

### Generation and Characterization of a Dyskinetic Mouse Model

The hemiparkinsonian mice were produced by unilateral injections of 6-OHDA targeted into the right MFB (Figure 1A). TH immunostaining revealed severe loss of striatal dopaminergic afferents in the mice with 6-OHDA lesions (Figure 1B). The quantifications revealed a >80% reduction of TH immunoreactivity in the dorsal striatum on the 6-OHDA-lesioned side compared to the non-lesioned side (Figure 1C; *P* < 0.001, paired two-tailed *t-*test). After a period of 3 weeks of levodopa/benserazide treatment, the AIM scoring tests revealed that all 6-OHDA-lesioned mice manifested LIDs. Figure 1D shows the time-sequential changes of AIMs in the dyskinetic mice, which were maximal 10–60 min after levodopa administration (i.e., “ON” period) and almost disappeared after 120 min.

Meanwhile, these dyskinetic mice also showed parkinsonian signs 6 h after levodopa administration (i.e., “OFF” period). The percentages of spontaneous rotations ipsilateral to the 6-OHDA-lesioned side (or right side) relative to the total rotations were significantly increased in mice with 6-OHDA lesions compared to mice without 6-OHDA lesions (Figure 1E; *P* < 0.01, Mann-Whitney U-test). Apomorphine-induced rotations contralateral to the 6-OHDA-lesioned side were also significantly increased in mice with 6-OHDA lesions as compared to mice without 6-OHDA lesions (Figure 1F; *P* < 0.01, Mann-Whitney U-test). Moreover, spontaneous use of hind limbs contralateral to the 6-OHDA-lesioned side was significantly decreased in mice with 6-OHDA lesions compared to mice without 6-OHDA lesions (Figure 1G; *P* < 0.05, Mann-Whitney U-test).

### Intrastriatal MMT Infusion Attenuates LIDs in 6-OHDA-Lesioned Mice

**Figure 2:**
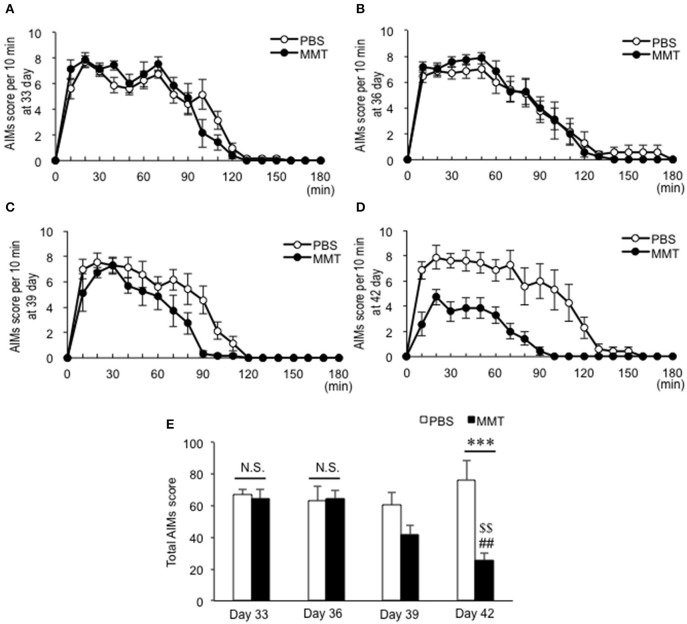
Effects of intrastriatal memantine (MMT) infusion on levodopa-induced abnormal involuntary movements (AIMs) in dyskinetic mice. **(A–D)** Time-sequential changes in the AIM scores every 10 min over a period of 180 min after the last levodopa administration in mice with intrastriatal infusion of PBS (*n* = 7) or MMT (*n* = 7) at day 33 **(B)**, day 36 **(C)**, day 39 **(D)**, and day 42 **(E)**. **(E)** Total AIMs score in mice with intrastriatal infusion of PBS or MMT at days 33, 36, 39, and 42. Data are means ± SEM. A significant effect of the MMT infusion treatment is found [two-way ANOVA: *F*_(group×*dose*)3,48_ = 5.856, *P* = 0.0017]. Bonferroni *post-hoc* test: PBS day 33 vs. MMT day 33, not significant (N.S.); PBS day 36 vs. MMT day 36, N.S.; and PBS day 42 vs. MMT day 42, ***P < 0.001. Significant differences in the severity of AIMs at days 33, 36, and 42 are found (Bonferroni *post-hoc* test: MMT day 33 vs. MMT day 42, ^##^*P* < 0.01; MMT day 36 vs. MMT day 42, ^$$^*P* < 0.01).

To determine if intrastriatal MMT infusion could reduce LIDs in the dyskinetic mice, we used the programmable and refillable iCBI systems equipped with iPRECIO^TM^ micro infusion pumps, which make it possible to perform quantitative pharmacology in single animals (20, 21). The iCBI devices (Figure 1H) with the infusion cannulas implanted into the dopamine-depleted striatum (Figures 1I–K) were placed at day 28 under the experimental protocols (Figure 1A). No severe damage of the tissue except for the cell clusters around the trace of infusion cannula, which may represent gliosis (Figure 1K), was observed. The levodopa/benserazide treatments were then restarted at day 31. Simultaneously, the MMT-treated dyskinetic mice received continuous intrastriatal infusion of MMT 0.28 ng/μl at a flow rate of 1.0 μl/h for the first 3 days (days 31–33), MMT 1.12 ng/μl at a flow rate of 1.0 μl/h for the next 3 days (days 34–36), MMT 4.48 ng/μl at a flow rate of 1.0 μl/h for the next 3 days (days 37–39), and MMT 17.93 ng/μl at a flow rate of 1.0 μl/h for the last 3 days (days 40–42). In parallel, the PBS-treated dyskinetic mice received PBS under the same protocol. Accordingly, the AIM scoring tests for the MMT- or PBS-treated mice were done at days 33 (Figure 2A), 36 (Figure 2B), 39 (Figure 2C), and 42 (Figure 2D). The two-way ANOVA on the total AIMs scores (Figure 2E) revealed a significant effect of the MMT infusion treatment [*F*_(group×*dose*)3,48_ = 5.856, *P* = 0.0017]. Compared to PBS infusion, MMT infusion significantly reduced AIMs at day 42 (Bonferroni *post-hoc* test: PBS day 42 vs. MMT day 42, ^***^P < 0.001). Moreover, a significant difference in the severity of AIMs was found at days 33, 36, and 42 (Bonferroni *post-hoc* test: MMT day 33 vs. MMT day 42, ^##^*P* < 0.01; MMT day 36 vs. MMT day 42, ^$$^*P* < 0.01). Thus, intrastriatal MMT infusion significantly attenuated levodopa-induced AIMs in a dose-dependent manner.

### Intrastriatal MMT Infusion Attenuates Parkinsonian Signs in 6-OHDA-Lesioned Mice

**Figure 3:**
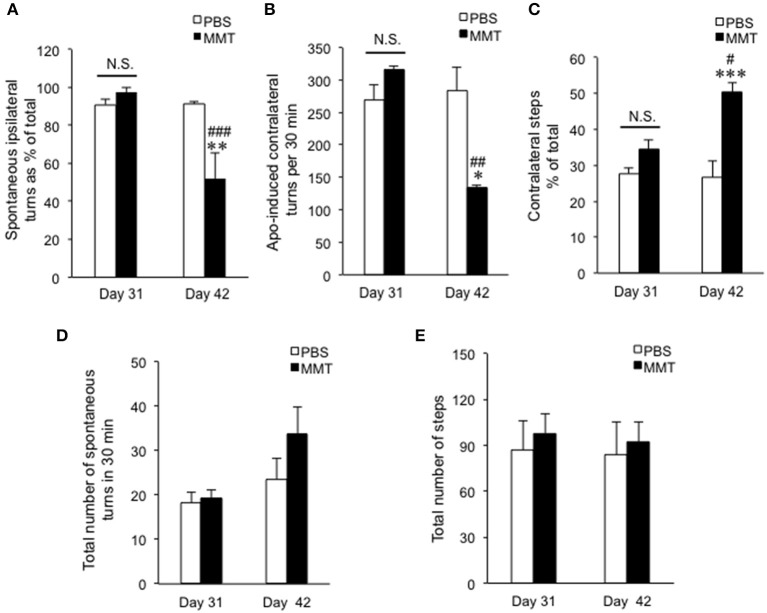
Effects of intrastriatal memantine (MMT) infusion on parkinsonian signs in the dyskinetic mice. **(A)** Spontaneous rotations ipsilateral to the 6-OHDA-lesioned side percent of the total in the groups of PBS infusion at day 31 (PBS day 31, *n* = 7), MMT infusion at day 31 (MMT day 31, *n* = 7), PBS infusion at day 42 (PBS day 42, *n* = 7), and MMT infusion at day 42 (MMT day 42, *n* = 7). Data are means ± SEM. A significant effect of MMT infusion [two-way ANOVA: *F*_(group×*dose*)1,24_ = 10.25, *P* = 0.0038] is observed. Bonferroni *post-hoc* test: not significant (N.S.); MMT day 31 vs. MMT day 42, **P < 0.01; PBS day 42 vs. MMT day 42, ^###^*P* < 0.001. **(B)** Counts of apomorphine-induced contralateral turns per 30 min in the groups of PBS day 31 (*n* = 6), MMT day 31 (*n* = 6), PBS day 42 (*n* = 6), and MMT day 42 (*n* = 6). Data are means ± SEM. A significant effect of MMT infusion [two-way ANOVA: *F*_(group×*dose*)1,20_ = 9.608, *P* = 0.0056] is found. Bonferroni *post-hoc* test: not significant (N.S.); MMT day 31 vs. MMT day 42, *P < 0.05; PBS day 42 vs. MMT day 42, ^###^*P* < 0.001. **(C)** Spontaneous contralateral hind limb steps as % of the total in PBS day 31 (*n* = 5), MMT day 31 (*n* = 5), PBS day 42 (*n* = 5), and MMT day 42 (*n* = 5). Data are means ± SEM. A significant effect of MMT infusion [two-way ANOVA: *F*_(group×*dose*)1,16_ = 7.891, *P* = 0.0126] is found. Bonferroni *post-hoc* test: not significant (N.S.); MMT day 31 vs. MMT day 42, ***P < 0.001; PBS day 42 vs. MMT day 42, ^#^*P* < 0.05. **(D)** Total number of turns in spontaneous rotations summed with both ipsi- and contra-lateral directions. Data are means ± SEM. No significance was observed [two-way ANOVA: *F*_(group×*dose*)1,24_ = 6.507, *P* = 0.0434]. **(E)** Total number of steps summed with both ipsi- and contra-lateral hind limbs. Data are means ± SEM. No significance was observed [two-way ANOVA: *F*_(group×*dose*)1,16_ = 0.3957, *P* = 0.5634].

We next determined if intrastriatal MMT infusion could also affect parkinsonian signs in the dyskinetic mice. For this purpose, behavioral tests for hemiparkinsonism were done 6 h after the last levodopa/benserazide treatments at days 31 and 42 (Figure 1A). Notably, the two-way ANOVA on the percentage of the spontaneous ipsilateral rotations relative to the total rotations (Figure 3A) revealed a significant effect of the MMT infusion treatment [*F*_(group×*dose*)1,24_ = 10.25, *P* = 0.0038]. MMT infusion significantly normalized the rotational asymmetry at day 42 (Bonferroni *post-hoc* test: MMT day 31 vs. MMT day 42, ^**^P < 0.01; PBS day 42 vs. MMT day 42, ^###^*P* < 0.001). The two-way ANOVA on the counts of apomorphine-induced contralateral turns (Figure 3B) also showed a significant effect of the MMT infusion treatment [*F*_(group×*dose*)1,20_ = 9.608, *P* = 0.0056]. MMT infusion significantly reversed the apomorphine-induced rotational behaviors at day 42 (Bonferroni *post-hoc* test: MMT day 31 vs. MMT day 42, ^*^P < 0.05; PBS day 42 vs. MMT day 42, ^##^*P* < 0.01). The two-way ANOVA on the counts of spontaneous contralateral hind limb stepping (Figure 3C) also showed a significant effect of the MMT infusion treatment [*F*_(group×*dose*)1,16_ = 7.891, *P* = 0.0126]. MMT infusion significantly normalized the % of contralateral hind limb steps at day 42 (Bonferroni *post-hoc* test: MMT day 31 vs. MMT day 42, ^***^P < 0.001; PBS day 42 vs. MMT day 42, ^#^*P* < 0.05). There was no significant difference between PBS and MMT groups in the total number of spontaneous turns (Figure 3D) nor of hind limb steps (Figure 3E). Thus, MMT infusion significantly improved not only AIMs but also parkinsonian signs in dyskinetic mice.

## Discussion

The long-term and repeated pharmacological overactivation of striatal dopamine receptors, which leads to an altered glutamatergic transmission at several nodes of the corticobasal ganglia circuit, is generally viewed as the principal cause for LIDs in patients with advanced Parkinson's disease. We provide herein experimental evidence that intrastriatal MMT infusion exerts therapeutic effects on both levodopa-induced AIMs and parkinsonian signs in mice with hemiparkinsonism caused by 6-OHDA. Although there is a difficulty to distinguish improvement of akinesia from induction of dyskinesia in rodent models of Parkinson's disease, and further studies using primate models are essentially required, our finding in this study corroborates the general thought that the overactivation of striatal NMDA receptors, which mediate a slow, Ca^2+^-permeable component of excitatory synaptic transmission (22), might generate LIDs and “OFF”-period motor deficits in Parkinson's disease (4, 5, 7, 23). Indeed, clinical observations have shown the positive therapeutic potential of the NMDA receptor antagonists amantadine (24) and MMT (25–28) in the treatment of LIDs and parkinsonian symptoms in patients with Parkinson's disease. However, the oral administration of NMDA antagonists, particularly amantadine, causes considerable noncerebral side effects, such as cardiovascular, gastrointestinal, and skin reactions (8, 26). It also incites cerebral adverse effects of nonstriatal origin, such as psychosis, hallucinations, and learning and memory deficit, because of the wide distribution of NMDA receptors in the brain (29). Considering the structural similarity between amantadine and MMT, these central and systemic side effects limit the usable dose of NMDA antagonists and reduce their clinical impact (30). A recent report of meta-analysis comparing multiple medicines in Alzheimer's disease patients showed that oral administration of MMT is safe enough (31). However, it is not approved for the treatment of Parkinson's disease patients yet. In a previous study, although single injection of MMT or amantadine did reduce LIDs in the 6-OHDA-lesioned rats, this antidyskinetic effect has disappeared by subchronic administration for a few days (32). It indicates that drug tolerance to MMT or amantadine developed during the treatment. A clinical study also reported that the beneficial effect of amantadine on dyskinesia was lost within 8 months (33). The underlying mechanism of MMT or amantadine tolerance, including whether it is caused by central or peripheral reasons, is still unknown. Given that the drug tolerance by repeated oral or systemic administration limits the therapeutic efficacy, it is worth developing an alternative drug delivery system, such as iCBI, despite of the risks, such as complications accompanying physical invasion.

The iCBI system makes it possible to deliver appropriately adjusted doses of desired drugs into specific brain regions. Therefore, intrastriatal infusion of NMDA antagonists through the iCBI system may provide higher therapeutic efficacy while reducing the adverse side effects of their oral or systemic administration in the treatment of LIDs. For the issue of drug tolerance, longer iCBI treatment of NMDA antagonists than this study should be examined. With the hope that programmable iCBI devices with a clinical application can be developed, we suggest the pharmacological deep-brain modulation as an alternative therapeutic approach for the treatment of Parkinson's disease and, potentially, other movement disorders.

## Data Availability Statement

The datasets generated for this study are available on request to the corresponding author.

## Ethics Statement

The animal study was reviewed and approved by The Institutional Animal Care and Use Committees of Tokushima University, Japan.

## Author Contributions

SG contributed to the conception and design of the study. MO, YZ, RT, JK, and SG contributed to the acquisition and analysis of data. MO, JK, and SG contributed to the drafting of a significant portion of the manuscript.

### Conflict of Interest

The authors declare that the research was conducted in the absence of any commercial or financial relationships that could be construed as a potential conflict of interest.
